# Clustering of Whole-Brain White Matter Short Association Bundles Using HARDI Data

**DOI:** 10.3389/fninf.2017.00073

**Published:** 2017-12-22

**Authors:** Claudio Román, Miguel Guevara, Ronald Valenzuela, Miguel Figueroa, Josselin Houenou, Delphine Duclap, Cyril Poupon, Jean-François Mangin, Pamela Guevara

**Affiliations:** ^1^Department of Electrical Engineering, Universidad de Concepción, Concepción, Chile; ^2^Neurospin, I2BM, CEA, Gif-sur-Yvette, France; ^3^APHP, Pôle de Psychiatrie, DHU PePsy, INSERM U955 Eq. 15 “Psychiatrie Translationnelle”, Université Paris Est, Créteil, France

**Keywords:** dMRI, HARDI, hierarchical clustering, short association bundles, white matter

## Abstract

Human brain connectivity is extremely complex and variable across subjects. While long association and projection bundles are stable and have been deeply studied, short association bundles present higher intersubject variability, and few studies have been carried out to adequately describe the structure, shape, and reproducibility of these bundles. However, their analysis is crucial to understand brain function and better characterize the human connectome. In this study, we propose an automatic method to identify reproducible short association bundles of the superficial white matter, based on intersubject hierarchical clustering. The method is applied to the whole brain and finds representative clusters of similar fibers belonging to a group of subjects, according to a distance metric between fibers. We experimented with both affine and non-linear registrations and, due to better reproducibility, chose the results obtained from non-linear registration. Once the clusters are calculated, our method performs automatic labeling of the most stable connections based on individual cortical parcellations. We compare results between two independent groups of subjects from a HARDI database to generate reproducible connections for the creation of an atlas. To perform a better validation of the results, we used a bagging strategy that uses pairs of groups of 27 subjects from a database of 74 subjects. The result is an atlas with 44 bundles in the left hemisphere and 49 in the right hemisphere, of which 33 bundles are found in both hemispheres. Finally, we use the atlas to automatically segment 78 new subjects from a different HARDI database and to analyze stability and lateralization results.

## 1 Introduction

One of the goals of white matter (WM) studies is the construction of an atlas of human brain connections, which is an important step toward the understanding of human brain function (Sporns, 2013). Human brain connectivity is extremely complex and variable across subjects; therefore, its description is still incomplete. Long association and projection bundles have been deeply studied due to their large size and stability across subjects. Therefore, a large number of studies have been carried out to describe, segment, and analyze these bundles. On the other hand, few studies about short association bundles of superficial white matter (SWM) exist. These bundles have a smaller size, an unknown structure, and a high intersubject variability, resulting in higher difficulties for their study. The analysis of SWM fibers is important to describe the human connectome to explain and predict human brain functions. Thus, their study can improve the knowledge of specific local connections by helping to understand functions and features that can be altered in some psychiatric disorders or neurological pathologies.

Diffusion magnetic resonance imaging (dMRI) is the preferred technique for the study of human brain white matter structure *in vivo* through the measurement of the restricted diffusion of water molecules (Basser, 1995). From dMRI, we can compute a diffusion local model that represents the main diffusion directions or local fiber orientations at each voxel. Using this information, tractography algorithms allow the reconstruction of the most probable fiber trajectories (Mori and Van Zijl, 2002). The most used diffusion local model is the diffusion tensor (DTI). This technique has allowed the study of long bundles on several neurological diseases, but the DTI model presents some limitations for the representation of fibers with different directions within a voxel. During the last 10 years, new techniques of dMRI with high angular resolution (HARDI) have been used, especially for research purposes. These images feature better quality and, together with a high-order local diffusion model (Tuch et al., 2002; Descoteaux et al., 2007), enable the identification of fiber crossing within a voxel, thus improving tractography results. With the adequate application of preprocessing and diffusion pipeline algorithms, whole-brain HARDI tractography data sets achieve a good representation of white matter bundle structure, including short association fibers. The obtained data sets can contain over a million fibers, even for deterministic tractography, when using all the WM voxels as seeds. Note that we use the term *fibers* to describe the three-dimensional polylines generated by a tractography algorithm. These do not represent real neural fibers, but an estimation of the main WM fiber pathways.

dMRI provides information about the integrity of the WM through the measurement of fractional anisotropy (FA). This index, along with other diffusion-based indices, allows the detection of differences in WM structure and infers connection characteristics of neurological diseases or psychiatric disorders. Known white matter bundles can be segmented in subjects for WM quantitative studies (O’Donnell and Pasternak, 2015). For example, differences were found in the corpus callosum, the cingulum, and the arcuate fasciculus for patients with bipolar disorder (Sarrazin et al., 2014). Other studies related changes in the cingulum with schizophrenia (Whitford et al., 2014) or in thalamic radiations with Alzheimer’s disease (Niida et al., 2013).

For the study of SWM, mainly two approaches have been used. First, previously published work has employed manual or automatic placement of regions of interest (ROI): Zhang et al. (2010) used a non-linear warping of a gray matter and white matter ROI atlas to extract the fibers that connect two gyri. With these results, probability maps of 29 short fibers from 20 subjects of a DTI database were created. This work was the first of its type, but no deep analysis of the bundles was performed and no bundle shape description was obtained. In he study by Pardo et al. (2013), the authors applied the same method to a HARDI database of 30 subjects (Schmitt et al., 2012), studying the variability of the obtained bundles.

The other group of works applies manual positioning of ROIs performed by an expert: Catani et al. (2012) used this approach to perform a detailed study of the frontoparietal association connections using HARDI data. Furthermore, a lateralization analysis of frontoparietal U-shaped tracts and premotor connections was performed using 12 subjects. Rojkova et al. (2016) applied the same method to segment the bundles described in this study, over a population of 47 subjects. The authors used a probabilistic representation of the bundles to study the differences in white matter due to age and education. Magro et al. (2012) characterized the short fibers of the brain central area using six ROIs, computed as a subdivision of the precentral and postcentral gyri. Next, fibers connecting pairs of regions were extracted from a whole-brain tractography. In the later study, by applying a similar strategy, the authors studied the connections within the primary motor cortex using the subdivision of the precentral gyri (Magro et al., 2014). By using a validation technique, Vergani et al. (2014) studied the connections of the supplementary motor area using postmortem dissections and dMRI. Region-based analyzes can be very precise on the extraction of specific short bundles when a manual delineation of ROIs is applied. However, this task requires an expert and is very time consuming; therefore, it is restricted to a small or medium-sized group of subjects and some brain regions. On the other hand, automatic parcellations of the cortex allow the study of whole-brain connections through the automatic extraction of the fibers traversing a group of regions. However, they use larger regions, thus leading to groups of fibers connecting two regions with inhomogeneous shape and a very high intersubject variability.

Other strategies are mainly based on fiber clustering, and use a distance metric between fibers. These have been extensively applied to known white matter bundles. O’Donnell and Westin (2007) was one of the first works that used clustering on a whole-brain tractography data set. The method applies spectral clustering to an affinity matrix, based on the mean closest point distance between fibers. Wassermann et al. (2010) proposed a hybrid approach to extract the most-known WM tracts. They model WM fibers as Gaussian processes, and use this model to define a distance metric between fibers. Their method then performs hierarchical clustering on the data and produces a dendrogram, which is then combined with *a priori* information given by a gray/white matter atlas to extract the most probable anatomical bundles. Visser et al. (2011) proposed the use of hierarchical clustering, applied to subsets of the data, to achieve good scalability. Guevara et al. (2012) applied an automatic intrasubject and intersubject clustering to 12 subjects of a HARDI database. The clusters present in at least half of the subjects were selected. Then a manual labeling was performed to create a multisubject atlas of deep white matter bundles. Several other works have been proposed to study those bundles (Durrleman et al., 2011; Garyfallidis et al., 2012; Yoo et al., 2015).

All the previous algorithms were designed to analyze known deep white matter bundles. Small differences between bundles did not need to be considered, as large bundles present a main core shape, composed by several sub-bundles, with variable spread extremities. In contrast, the analysis of short association bundles requires more stringent similarity measures, to discriminate bundles connecting different small cortex regions. Only very similar fibers, with the same shape along the entire bundle, must be regrouped. The first attempt of clustering short association fibers was done by Guevara et al. (2012), leading to the creation of an atlas of short association fibers of the left hemisphere. A total of 47 short bundles were found, with medium to high reproducibility across subjects. A normalization term was used to take into account fiber length. However, results were very preliminary, with a high inhomogeneity within each cluster and were inferred from only 12 subjects.

Recently, Guevara et al. (2017) proposed a hybrid method for the reproducibility study of short association bundles. For each subject, subtractograms connecting pairs of gyri were extracted. Then, intrasubject shape-based fiber clustering performed a compression of each subtractogram into a set of bundles. Finally, for each pair of gyri, a match of the bundles across subjects was found using intersubject clustering. The method found a total of 100 bundles for the whole brain, with medium to high reproducibility across subjects. From the point of view of the construction of an SWM bundle atlas, results from the study by Guevara et al. (2017) are interesting, because they used a large and high-quality database and performed validation using two groups of subjects. The advantage of this method is that it can find reproducible bundles across the whole brain, including those with moderate reproducibility. However, this method relies on the cortical parcellation being applied to the fibers. All the subsequent steps, mainly based on clustering algorithms, are applied individually to each group of fibers connecting two gyri. Errors in cortical parcellation and intersubject registration will impact the final bundles, in particular those localized in the frontiers of the gyri.

To overcome this issue, we propose a method for the study of SWM based on the intersubject clustering of whole-brain short white matter fibers. This approach has also the advantage of naturally including in the study the connections within the gyri. In contrast to the study by Guevara et al. (2012), the method was adapted to short fibers, with the capacity to deal with a larger group of subjects and the WM structural complexity of a high-quality database (Schmitt et al., 2012), in a reasonable time. To deal with a large number of fibers and reduce dimensionality, the analysis is applied to the short centroids of preclustered fibers (Guevara et al., 2011b). First, the maximum distance between corresponding points is computed between all centroids. Next, an average link hierarchical clustering is performed over the centroids, based on an affinity graph. Then, to obtain tight clusters, an adaptive partition of the resulting hierarchical tree is performed, according to the maximum distance between the centroids of a cluster. Resulting clusters are then evaluated and validated. A validation was also performed by comparing the results from the application of the method to pairs of independent groups of 27 subjects following a bagging with a total of 74 subjects. In addition, the influence of the intersubject registration algorithm was evaluated. To achieve that goal, the method was applied using both affine registration and a non-linear registration algorithms based on the diffusion tensor (Zhang et al., 2006). Results from non-linear registration were found to be more reproducible and were used to create an atlas containing the most stable bundles. Finally, the atlas was used to segment the SWM bundles of 78 new subjects and study the reproducibility and lateralization of short bundles.

## 2 Materials and Methods

### 2.1 Tractography Data Sets

#### 2.1.1 Development Data Sets

Development and segmentation data sets were used. All the subjects provided an informed written consent, and the data acquisition was performed with the approval of the local ethical committees. We used seventy-four healthy subjects (23.6 ± 5.2 years old; 43 males, 31 females; 71 right handed and 3 left handed), from a high quality HARDI database (Schmitt et al., 2012). Scans were acquired on a Tim Trio 3 T MRI system with a 12-channel head coil (Siemens, Erlangen), and the MRI protocol included the acquisition of a T1-weighted data set using an MPRAGE sequence (160 slices; TH = 1.10 mm; TE/TR = 2.98/2,300 ms; TI = 900 ms; flip angle FA = 9; matrix = 256 × 240; voxel size = 1 mm × 1 mm × 1.1 mm; RBW = 240 Hz/pixel), a B0 field map, and a SS-EPI single-shell HARDI data set along 60 optimized diffusion weighted directions, b = 1,500 s/mm^2^ (70 slices; TH = 1.7 mm, TE = 93 ms; TR = 14,000 ms; FA = 90; matrix = 128 × 128; voxel size = 1.71875 mm × 1.71875 mm × 1.7 mm; RBW = 1502 Hz/pixel; echo spacing ES = 0.75 ms; partial Fourier factor PF = 6/8; GRAPPA = 2; total scan time = 16 min and 46 s).

The data were preprocessed using BrainVISA/Connectomist-2.0 software (Duclap et al., 2012). They were preliminary corrected for artifacts (eddy currents, susceptibility effects, spikes, and noise), and outliers were also removed. Then, the analytical Q-ball model (Descoteaux et al., 2007) was computed. Whole-brain regularized streamline deterministic tractography (Perrin et al., 2005) was performed on the diffusion-weighted (DW) native space, using a T1-based propagation brain mask (Guevara et al., 2011a), with a forward step of 0.2 mm and a maximum curvature angle of 30. The mask was used for seeding (one seed per voxel at T1 resolution) and to define the space where fibers were tracked. The propagation mask is constructed to have a good WM mask, in particular subcortical white matter, leading to a good reconstruction of short SWM fibers. Resulting tractography data sets present an average of one million fibers per subject, each with a length between 20 and 300 mm.

**Figure 1:**
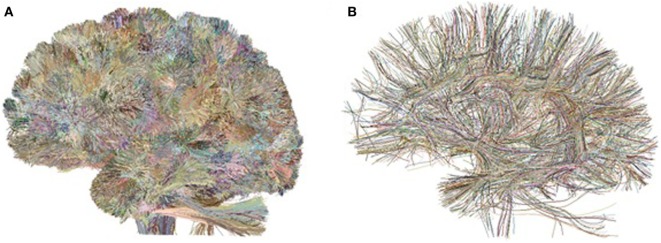
Preclustered data sets: example of intrasubject fiber clusters **(A)** and their centroids **(B)** for the left hemisphere of Subject 1.

Fibers were then processed using intrasubject clustering (Guevara et al., 2011b), to remove outliers and reduce the data dimensionality. The clustering generates two tractography data sets per subject (Figure 1): the cluster data set, consisting of compact fascicles of similar fibers, and the cluster centroid data set, containing a representative fiber for each cluster, resampled with 51 equidistant points. The data sets present an average of 5,300 clusters per subject. All the fibers are in T2 subject space, i.e., in each individual diffusion-weighted space. We term these data sets the *preclustered data*.

#### 2.1.2 Segmentation Data Sets

Seventy-eight subjects from a high-quality HARDI database were used for the segmentation of the SWM bundles obtained with the method. Scans were acquired on a Tim Trio 3TMRI system with a 12-channel head coil (Siemens, Erlangen), and the MRI protocol included the acquisition of a T1-weighted data set using the same protocol employed for the main database, a B0 field map, and a SS-EPI single-shell HARDI data set along 60 optimized diffusion weighted directions, b = 1,400 s/mm^2^ (70 slices; TH = 2.0 mm, TE/TR = 92/9,300 ms: FA = 90; matrix = 128 × 128; RBW = 1,502 Hz/pixel; echo spacing ES = 0.75 ms; partial Fourier factor PF = 6/8; GRAPPA = 2; voxel size = 2.0 mm × 2.0 mm × 2.0 mm).

The data were preprocessed using BrainVISA/Connectomist-2.0 software (Duclap et al., 2012) using the same steps as for the main database up to the computation of the tractography data set. Tractography data sets were calculated in T2 subject space, but an affine normalization to Talairach space was applied to each data set for bundle segmentation purposes.

### 2.2 Clustering-Based SWM Fiber Bundle Identification

**Figure 2:**
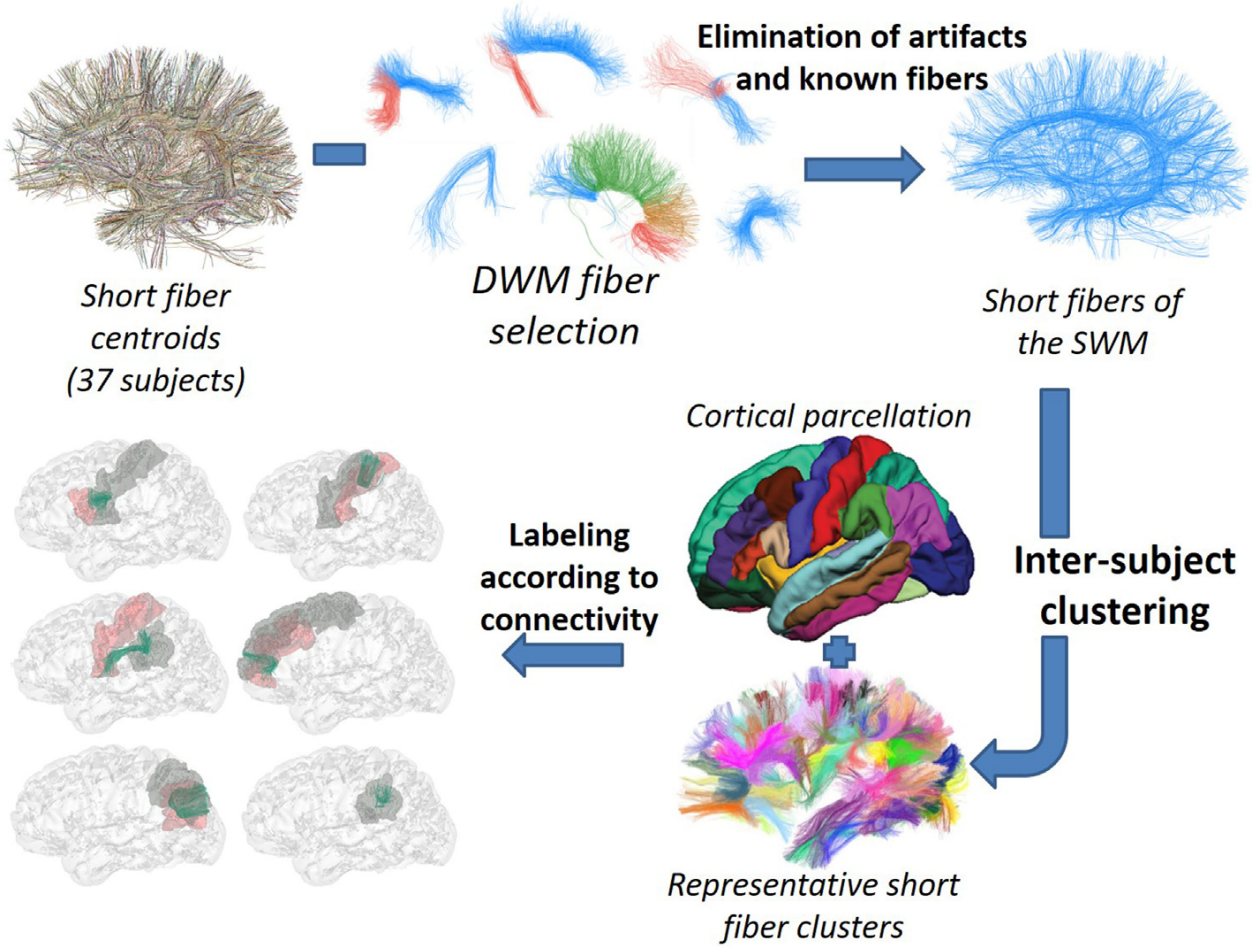
Schematization of the SWM bundle identification process. © 2016. Reprinted, with permission, from IEEE 38th Annual International Conference of the Engineering in Medicine and Biology Society (EMBC), 2016, p. 5545–9.

Our goal was to develop a method for the identification of the most reproducible SWM bundles of the whole brain, based on intersubject clustering, and adapted to high-quality databases. The method, depicted in Figure 2, begins with a selection of short centroids, to reduce the number of elements and focus the analysis on short association fibers. Next, the selected centroids from a group of subjects in Talairach space are clustered using a fiber distance metric. Only reproducible clusters are carried onto the next step. An anatomical labeling using cortical parcellations is applied. Finally, only stable connections, with a significant main connection for most of the subjects, are selected. The rest of this section describes each of these steps in detail.

#### 2.2.1 Short SWM Fiber Selection

Short centroids, with a length between 35 and 85 mm, were selected from the tractography data sets of the development database. Most of these centroids belong to superficial short fibers. Length selection was derived from the observation of bundles with different lengths, using the preclustered data. Fibers shorter than 35 mm were discarded, because their shape is very variable and difficult to analyze. In most cases, these fibers are artifacts and do not represent structured bundles. Even in the cases were the fibers are regrouped into bundles, these belong to a short association bundle that is better represented by longer fibers. We selected an upper threshold of 85 mm for fiber length, because short U-shaped fibers do not exceed this size.

Using a multi-subject deep white matter (DWM) bundle atlas (Guevara et al., 2012), the data are preprocessed to remove short centroids that can be part of known bundles. This occurs because some DWM bundles are composed in part of short fibers. Another cause is the presence of artifacts that can be generated during tractography, e.g., fibers belonging to long bundles that were cut by the mask. This phenomenon occurs in the periphery of the WM mask, where voxels are difficult to label due to the partial volume effect. Hence, cut bundles or badly reconstructed bundles are mixed with short association fibers. To remove all these fibers, which could be very similar to some fibers in deep white matter bundles, we compare the short centroids with selected bundles of the DWM bundle atlas. The DWM bundles chosen for comparison are the short bundles of the arcuate fasciculus, i.e., the anterior and posterior segments, uncinate fasciculus, short and temporal fibers of the cingulum, fornix, posterior and inferior thalamic radiations, and a cut version of corpus callosum fibers. Bundles such as the inferior frontooccipital fasciculus or the corticospinal tract are composed of only long fibers; therefore, they were not used. The corpus callosum is of particular case: our method is applied to both hemispheres separately, so the corpus callosum fibers were cut across the interhemispheric plane to obtain the left and right portions of this bundle. The DWM atlas is in the Talairach space; therefore, for this comparison, the short centroids were transformed to this space using an affine normalization.

The method computes a distance matrix between the fibers from the two data sets, i.e., the selected DWM bundles and short centroids from all the subjects. The distance metric used to compare each pair of fibers is the maximum of the Euclidean distances between corresponding points (*d_ME_*), which is calculated as shown in equation (1):

(1) $$d_{ME}(A,B)=min({max}_{i}∥a_{i}-b_{i}∥,{max}_{i}∥a_{i}-b_{\left(N_{p}-i\right)}∥),$$

where *a_i_* and *b_i_* are the positions of the corresponding points of a pair of fibers *A* and *B*. The distance is symmetrized, by taking the minimum of the two possible directions of the fibers, as described by O’Donnell and Westin (2007). This distance was chosen because it is more restrictive than the minimum of the mean distances between closest points (O’Donnell and Westin, 2007) or between corresponding points (Garyfallidis et al., 2012). Centroids are considered similar to the selected DWM bundles when the distance between the centroids and the DWM fibers is inferior to a threshold equal to 10 mm. This value is very restrictive for long bundles, so only centroids very similar to DWM fibers are discarded. Because the bundles selected for this process do not contain unknown short fibers, there is no risk of discarding short bundle candidates. To speed up the calculation, only a random sampling of 20% of the selected atlas fibers is used, representing about 400 fibers per bundle. This is possible because DWM bundles are very dense and very stable across subjects, so samples from different subjects are very similar.

#### 2.2.2 Intersubject Clustering

**Figure 3:**
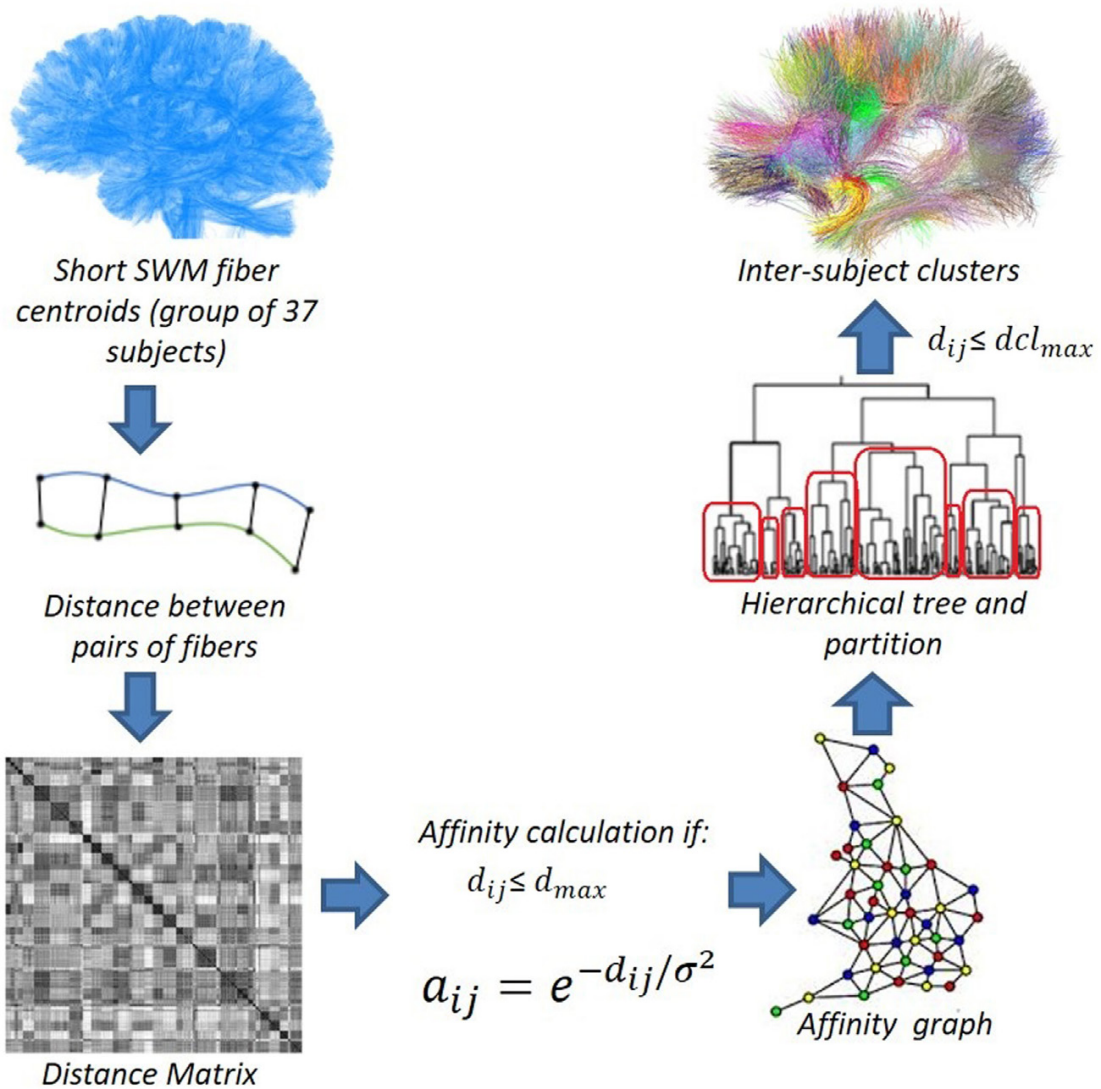
Data flow of the intersubject fiber clustering method. © 2016. Reprinted, with permission, from IEEE 12th International Symposium on Biomedical Imaging (ISBI), 2015, p. 440–4.

By using the short SWM centroids, we apply intersubject hierarchical clustering to a group of subjects (Figure 3), where the centroids are aligned in a common space. In this work, we used two types of normalization methods: affine registration to Talairach space and non-linear registration to DTI-TK “IXI aging DTI template.” More details about these registration methods are described in Section 2.4.

We compute a distance matrix *M* for all the pairs of SWM centroids using the distance *d_ME_*. Then, from the matrix, we compute an affinity graph. The affinity is defined as $a_{ij}={\text{e}}^{-d_{ij}/\sigma^{2}}$ (O’Donnell and Westin, 2007), where *d_ij_* is the distance between the elements *i* and *j*, and *σ*^2^ is a parameter that defines the similarity scale (60 mm). The affinity computation is performed for each pair of fibers with a distance smaller than a defined maximum distance (*d_clmax_*). This processing allows us to significantly reduce the clustering processing time, as only about 5% of the edges are included in the affinity graph.

We then run an average-link hierarchical agglomerative clustering algorithm on the affinity graph. This algorithm computes a hierarchical tree (or dendrogram) with all the cluster fusions. The original implementation of the algorithm is included in the nipy Python library.^1^ It can deal with big data sets, taking advantage of the sparcity of the affinity graph.

We then compute an adaptive partition of the hierarchical tree, according to a maximum distance *d_clmax_* between the centroids of a cluster. The analysis starts from the top node and traverses the entire tree, selecting nodes to be analyzed and storing their indices in a queue. For each of these nodes, the algorithm computes the maximum pairwise distance between all its centroid descendants. If this distance is smaller than or equal to the maximum distance *d_clmax_*, the centroids are grouped into a cluster. Otherwise, the two children nodes are added to the queue to be analyzed. Typical values for *d_clmax_* in fiber data vary between 15 and 45 mm.

To cluster a large number of elements (more than 50,000 centroids), we implemented a version of the clustering algorithm that does not store the entire distance matrix *M* in memory, but rather directly computes the affinity graph using the distance threshold *d_clmax_*. Algorithm 1 shows how the dendrogram is created from the list of fibers and the list of affinities between them.

**Algorithm 1:** Clustering between *N* fibers with *M* affinities and nC unconnected elements.

**function** Clustering elements, affinities
**for** i := 1 to N - nC **do**
Find the highest affinity between a pair of elements and use it as a new node of the dendrogram
Remove the affinity between the selected pair of elements
Merge the clusters associated to each of the elements
Update the affinities using the average-linkage criterion
**end for**
**return** dendrogram
**end function**

Later, the partition computation stage uses a sparse representation of the matrix *M*, containing only the distances smaller than the maximum distance (*d_clmax_*). Once the partition is computed, it is analyzed to verify the number of subjects involved in each cluster. Bundles that are representative of the population, that is, that exist in most subjects, are identified. We use a strict selection criterion, considering as representative only the bundles present in at least 75% of the subjects. Note that in some cases, short association fibers are not reconstructed due to partial volume effects or tractography artifacts. Also, it is possible to miss bundles in some subjects due to intersubject variability or normalization errors.

### 2.3 Robust Atlas Creation

#### 2.3.1 Identification of Similar Bundles between Two Different Groups of Subjects

The proposed method was applied to two independent groups of N subjects from the same database to create a robust atlas. We implemented a comparison algorithm between bundles from different groups to identify the similar bundles across both groups of subjects.

First, the intersection between similar bundles is computed. For each pair of bundles, composed by bundles from different groups, the distance between all the fibers from each bundle was calculated using distance *d_ME_*.

For each bundle, we compute the number of fibers presenting at least one close fiber from the other bundle, with a distance smaller than 5 mm. The number of fibers of a bundle that match this criterion is used as a measure of the intersection between bundle sets. Pairs of bundles with an intersection percentage higher than 50% are considered similar, as they present an important overlap between them.

#### 2.3.2 Atlas Creation

All the processing, including *clustering-based SWM fiber bundle identification* (Section 2.2) and the *identification of similar bundles between two different groups of subjects* (Section 2.3.1), is applied to the centroids of a set of subjects, divided into two groups of N subjects.

After the comparison between the results of the two groups of N subjects, the pairs of similar bundles from the two groups are fused. In some cases, two very similar bundles could be separated, because of small differences in fiber shape. We chose to fuse them, because too much fine granularity is not desirable for short association bundles due to intersubject variability.

#### 2.3.3 Automatic Labeling

The representative bundles obtained by the clustering algorithm are analyzed using anatomical information to label the bundles according to the regions that they connect. Due to their shape, short association bundles (U-fibers) connect two regions. Therefore, the labels indicate the pair of ROIs connected by each bundle.

A parcellation of the cerebral cortex calculated using FreeSurfer^2^ is employed. This is based on the major gyri and sulci, according to the Desikan-Killiany atlas (Desikan et al., 2006), that has 34 parcels per hemisphere (see Table 1). From each subject parcellation, an ROI image is generated. For the analysis, the centroids of reproducible bundles are transformed to individual T1 referential. An oversampling is first applied to every centroid to ensure that at least one point of the centroid extremities is located in the connected cortical ROIs. The algorithm detects the two ROIs connected by each centroid of each bundle. This analysis is performed separately for each subject. After that, the results for each bundle are analyzed together for all the subjects in the group.

**Table 1:** ROIs of the Desikan–Killiany atlas (Desikan et al., 2006) and labels.

ROI	Abbreviation	ROI	Abbreviation
Bankssts	B	Pars opercularis	Op
Caudal anterior cingulate	CACg	Pars orbitalis	Or
Caudal middle frontal	CMF	Pars triangularis	Tr
Corpus callosum	CC	Pericalcarine	PerCa
The cuneus	Cu	Postcentral	PoC
Entorhinal	En	Posterior cingulate	PoCg
Fusiform	Fu	Precentral	PreC
Inferior parietal	IP	Precuneus	PreCu
Inferior temporal	IT	Rostral anterior cingulate	RoACg
Isthmus cingulate	IstCg	Rostral middle frontal	RoMF
Lateral occipital	LO	Superior frontal	SF
Lateral orbito frontal	LOrF	Superior parietal	SP
Lingual	Lg	Superior temporal	ST
Medial orbito frontal	MOrF	Supramarginal	SM
Middle temporal	MT	Frontal pole	FPol
Parahippocampal	PaH	Temporal pole	TPol
Paracentral	PaC	Transverse temporal	TrT
Insula	Ins		

In most cases within a bundle, subsets of centroids connect different pairs of neighboring ROIs. Also, sometimes, the centroids connect different areas of one ROI. After a visual analysis of the results, we concluded that the presence of different connections within a cluster could be due to clustering, registration, or cortical parcellation errors, but in most cases, a main and reproducible connection between two ROIs could be found. For each bundle, the percentage of centroids connecting each ROI pair, and also the number of subjects presenting each connection, was calculated. Finally, the most stable connection for each bundle is selected and used as label.

#### 2.3.4 Interhemispheric Correspondence

To automatically assign the same labels to the common bundles between the left hemisphere (LH) and the right hemisphere (RH), an interhemispheric bundle correspondence analysis was applied to the atlas bundles.

For the right bundles, their symmetric horizontal reflection is calculated to obtain their position in the LH. With bundles from both hemispheres in the same space, the intersection between similar bundles is calculated using the same processing described in Section 2.3.1.

Pairs of bundles from different hemispheres with an intersection percentage higher than 50% are considered common bundles between hemispheres. Similar bundles in both hemispheres are renamed to have a common label.

### 2.4 Registration Method Analysis

Due to the morphology of each brain, linear registration could be insufficient for the application of intersubject clustering methods. Non-linear registration can potentially improve the alignment of the subjects and hence the results. Therefore, to compare the behavior of our robust atlas creation method (Section 2.3.1) with different kinds of normalization methods, we applied it to two centroid data sets consisting of two groups of 37 subjects aligned using affine registration to Talairach space, and the same two groups aligned using a non-linear registration method.

For linear registration, preclustered centroid data sets were transformed to Talairach space, using an affine normalization, based on AC-PC alignment. For non-linear registration, we used DTI-TK,^3^ a method based on the features of the diffusion tensor (Zhang et al., 2006). For the alignment, the “IXI aging DTI template” was employed. First, an affine registration is applied to the DTI image of each subject, obtaining the affine aligned image and the corresponding affine transformation. Next, a diffeomorphic registration on the affine aligned image to the template is calculated, obtaining the diffeomorphic aligned image and the corresponding deformation field. Preclustered centroid data sets were transformed to the template space using the calculated affine transformation and deformation field, applied to each centroid coordinate.

### 2.5 Automatic Segmentation of SWM Bundles

The created atlas contains the most stable bundles, common to both groups of subjects. To evaluate the reproducibility of the bundles composing the atlas, these were used for the automatic segmentation of 78 new subjects from the segmentation database.

The segmentation method calculates the distance *d_ME_* (equation (1)) between each atlas bundle centroid and each fiber in the subject from the segmentation data set, normalized to the difference between the atlas centroid and the subject fiber lengths (Labra et al., 2017):

(2) $$\begin{array}{l} d_{me_{n}}=\{\begin{array}{cc} d_{me}(A,B)+dnf, & \text{if}\;dnf>0\\ d_{me}, & \text{otherwise} \end{array}\\ \text{with}\;dnf={\left(\frac{abs(l_{A}-l_{b})}{max(l_{A},l_{B})}+1\right)}^{2}-1, \end{array}$$

where *dnf* is a normalization factor that penalizes the length difference between the atlas centroids and the subject fibers. A restrictive threshold was used to label the short association bundles, thus selecting only the fibers that are very similar to the atlas bundles. We set a threshold between 6 and 8 mm, according to the length of the bundles.

The symmetrized atlas was used for segmentation and to analyze short association bundle lateralization. The remaining reproducible atlas bundles, present only in one hemisphere, were also segmented.

#### 2.5.1 Lateralization Index

For bundles segmented in both hemispheres, a lateralization analysis was performed. The volume of each bundle was calculated using a mask, with a voxel size of 2 mm × 2 mm × 2 mm. A bundle image was first constructed, with voxels counting the number of fibers in the bundle passing across them. Then, the bundle image was binarized, considering the voxels with more than one fiber. The volume of each bundle was calculated as the number of voxels in the bundle mask for each segmented subject. The lateralization index (LI) of a bundle was calculated using the formula: LI = (Right volume − Left volume)/(Right volume + Left volume), as used by Catani et al. (2012). LI is a number between −1 and 1, where negative values indicate a left lateralization.

## 3 Results

This section describes the results of our method in three parts: first, the method was applied to test the intersubject clustering algorithm, analyze the robust atlas creation method, and to select the normalization strategy (Section 3.1). Then, a validation method was performed, leading to the creation of a final atlas (Section 3.2). Finally, automatic segmentation was applied to test the reproducibility of the final atlas of SWM bundles (Section 3.3).

### 3.1 Robust Atlas Creation Results

To analyze the registration method, the algorithm was applied to two independent groups of 37 subjects from the development HARDI database, using both types of registration. Each hemisphere was analyzed separately.

Each subject hemisphere in our data set contains on average 2,345 centroids, from which 1,131 centroids on average correspond to short fibers (35–85 mm). After the comparison between the selected DWM atlas bundles, around a 17% of the centroids were discarded, leading to a mean of 939 centroids per hemisphere, per subject. This results in around 35,000 centroids per group in each hemisphere.

#### 3.1.1 Clustering-Based SWM Fiber Bundle Identification

We applied intersubject clustering using different values for the maximum distance *d_clmax_*, between 15 and 45 mm. The total number, shape, and size of the clusters were analyzed, as well as the reproducibility of the bundles. We looked for bundles that were large enough to ensure good reproducibility, but also preserve good shape similarity within the fibers of the bundle. The best results were obtained with a value of *d_clmax_* = 30 mm to compute the clusters. After the computation of the dendrogram, the partition stage of the algorithm selects reproducible clusters with centroids that belong to at least 75% (28 of the 37 subjects in the particular case of the atlas used for the normalization analysis).

While most of the stages of the algorithm are largely implemented as Python scripts, we wrote an optimized C++ version of the dendrogram construction algorithm depicted in Algorithm 1, which accounts for most of the execution time. While Python offers enhanced flexibility and productivity, our C++ version of the algorithm allows us to improve performance using advanced code optimizations and more efficient memory management. In fact, on our test machine, the Python code supports a maximum of 15,000 centroids, while the C++ implementation was successfully executed with up to 150,000 centroids.

**Figure 4:**
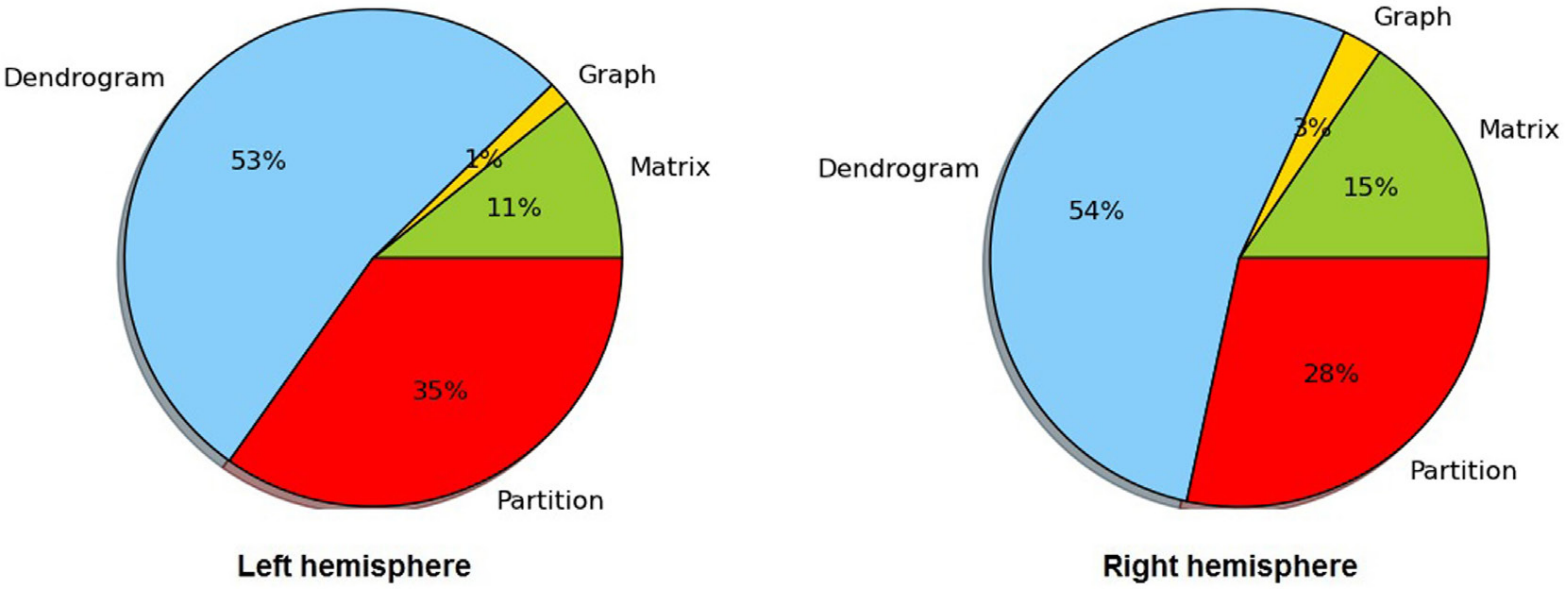
Distribution of the execution time for dendrogram construction in both hemispheres of Group 1.

Table 2 lists the number of centroids and the execution times for each step of the intersubject clustering process, for each group and each hemisphere, using non-linear registration. The process is divided into the following steps: distance matrix calculation, affinity graph construction, dendrogram calculation, and dendrogram partition. The entire process takes between 2.6 and 3.4 h on our test machine. Figure 4 more clearly shows the distribution of the clustering execution time for each hemisphere in Group 1. Even using the optimized C++ implementation, the construction of the dendrogram is responsible for more than 50% of the execution time in all cases, followed by the partition stage, which accounts for about one third of the total time. We run our experiments on a 64-bit workstation with an Intel i7-4770 quad core processor at 3.4 GHz and 16 GB of DDR3 memory.

**Table 2:** Execution time for all stages of the clustering process.

		Centroids	Matrix (min)	Graph (min)	Dendrogram (min)	Partition (min)	Total (min)
Group 1	LH	34,375	22	3	108	71	204
	RH	35,251	24	4	83	44	155

Group 2	LH	34,571	23	2	110	46	181
	RH	34,539	22	5	108	43	178

To determine the anatomical labeling, we calculated the percentage of fibers connecting each ROI pair for each bundle and the number of subjects containing the connection. Bundles with a strong connection were selected and named. Discarded bundles with weak connections have extremities localized on the limits of several regions.

#### 3.1.2 Analysis of the Method Behavior and Normalization Selection

Table 3 shows the number of bundles found for each hemisphere and each group, using both registration methods. For non-linear registration, we obtained about 60% more fascicles than using linear registration. In addition, bundles from non-linear registration are more dense and, on average, more reproducible across subjects. No significant difference was found in the number of bundles from both hemispheres.

**Table 3:** Number of clusters for each hemisphere and each group of subjects.

	Linear		Non-linear	
	Group 1	Group 2	Group 1	Group 2
LH	98	97	157	169
RH	96	97	167	164

We calculated the similarity between each pair of bundles from different groups of subjects, using a restrictive value for the maximum mean distance *d_Th_*. All the bundles under this threshold were considered very similar. Two different thresholds were used for this analysis: 7 and 10 mm, and Table 4 summarizes our results. By using *d_Th_* = 10 mm, we obtained a large number of similar bundles, especially using non-linear registration. However, for some cases, bundles did not present a high similarity between groups. By using *d_Th_* = 7 mm, we obtained better results for non-linear registration, but unsatisfactory results for linear registration: only 2 similar bundles between groups in each hemisphere were found for linear registration, while for non-linear registration, we obtained 55 and 42 similar bundles for the left and right hemisphere, respectively. These results are due to a better alignment of fibers using non-linear registration, which produces more reproducible bundles across subjects using the same intersubject clustering method.

**Table 4:** Number of similar bundles from the intergroup comparison, for linear and non-linear registration.

	Linear		Non-linear	
	*d_Th_* = 10	*d_Th_* = 7	*d_Th_* = 10	*d_Th_* = 7
LH	40	2	91	55
RH	31	2	97	42

To identify the differences and similarities between the bundles obtained from both registration methods, we compared the results from intergroup comparison, using *d_Th_* = 7 mm for non-linear registration and *d_Th_* = 10 mm for linear registration. A comparison using the smaller threshold for both methods is not possible as only two bundles were found for linear registration. Also, a comparison using the higher threshold is not useful as bundles for non-linear registration are better for *d_Th_* = 7 mm. Pairs of bundles with a mean distance smaller than 25 mm were computed, which is higher than the threshold used within a single registration method, because bundles obtained with different registration methods present a more heterogeneous structure. From these results, we can observe that about 60% of the bundles can be identified with both registration methods, 31 for the left hemisphere and 32 for the right hemisphere. However, the bundle reproducibility and homogeneity are better for non-linear registration. The other 40% of the bundles could be identified only with non-linear registration, 17 for the left hemisphere and 22 for the right hemisphere, highlighting the advantages of the non-linear method.

On the basis of registration results described above, we chose to use only non-linear registration in the next steps of the method.

### 3.2 Validation Method and Final Atlas Construction

To perform a better validation of the results and obtain a measure of bundle reproducibility, we embedded our method inside a bagging strategy (bootstrap aggregating) performed over the ARCHI database (74 subjects). For the atlas creation, the robust atlas creation method uses two independent groups of subjects. Hence, for the validation, this approach was repeated 10 times, employing sets of 54 subjects sampled randomly from the ARCHI database, split into two groups of 27 subjects. This processing led to 10 SWM bundle atlases for each hemisphere.

To build the final atlas, first a bundle centroid was calculated for each bundle. Then, for each hemisphere, our hierarchical clustering was performed over the bundle centroids from the 10 atlases. For cluster selection, a maximum distance between centroids equal to 30 mm was used (this distance value is the same used for the robust atlas creation method). Only bundles present in at least 8 of 10 tests were considered in the final atlas. The obtained bundles were then named using the automatic labeling strategy, based on the pair of ROIs connected by each bundle. Only bundles with a strong connection, present in at least 50% of the fibers, were selected.

Finally, interhemispheric correspondence was automatically calculated. To identify these common bundles between the two hemispheres, the similarity between the left atlas bundles and the reflected right atlas bundles was calculated by measuring the intersection between bundles. Pairs of bundles with more than 50% of similar fibers, using a distance threshold smaller than 5 mm, were considered common bundles between hemispheres.

**Figure 5:**
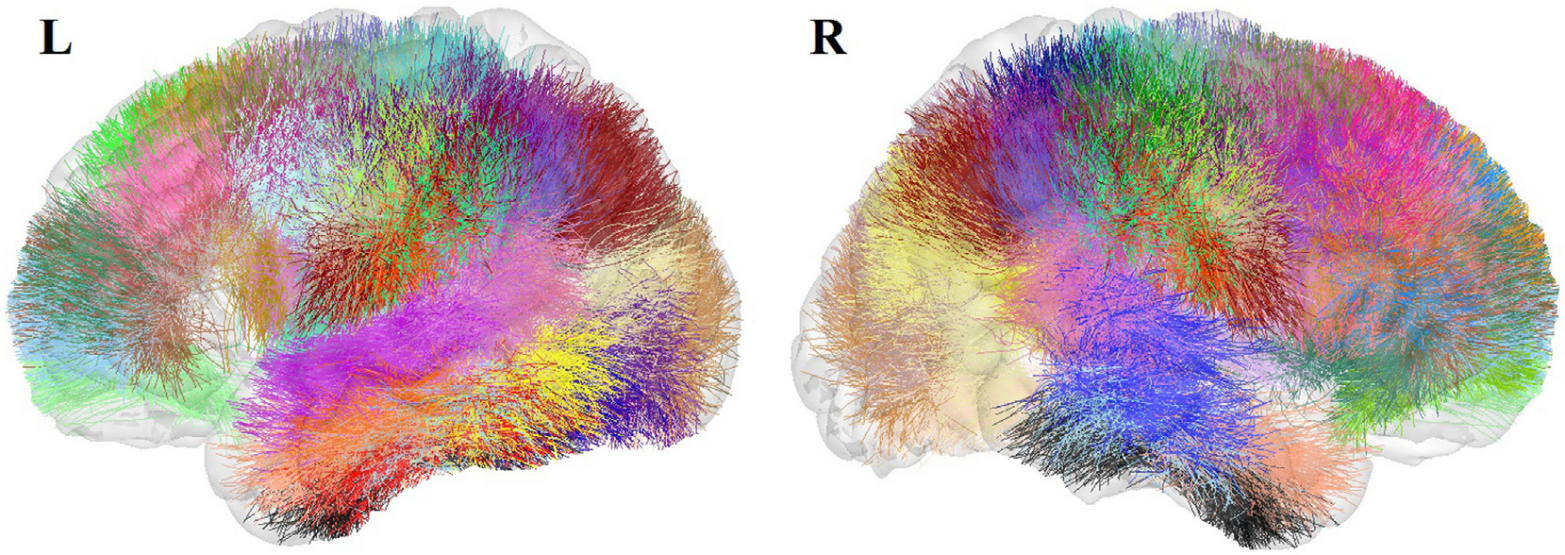
Atlas of the superficial white matter, composed of 44 bundles in the LH and 49 in the RH.

An atlas with a total of 93 bundles was finally obtained (see Figure 5). The atlas contains 44 bundles in the left hemisphere, 49 bundles in the right hemisphere, and 33 bundles common to both hemispheres.

For lateralization analyzes, a symmetrized version of the final atlas was created with the common bundles in both hemispheres. Common bundles were fused to create the symmetrized left bundles. Then, these bundles were reflected to the RH to create the symmetrized right bundles and thus construct the atlas.

**Figure 6:**
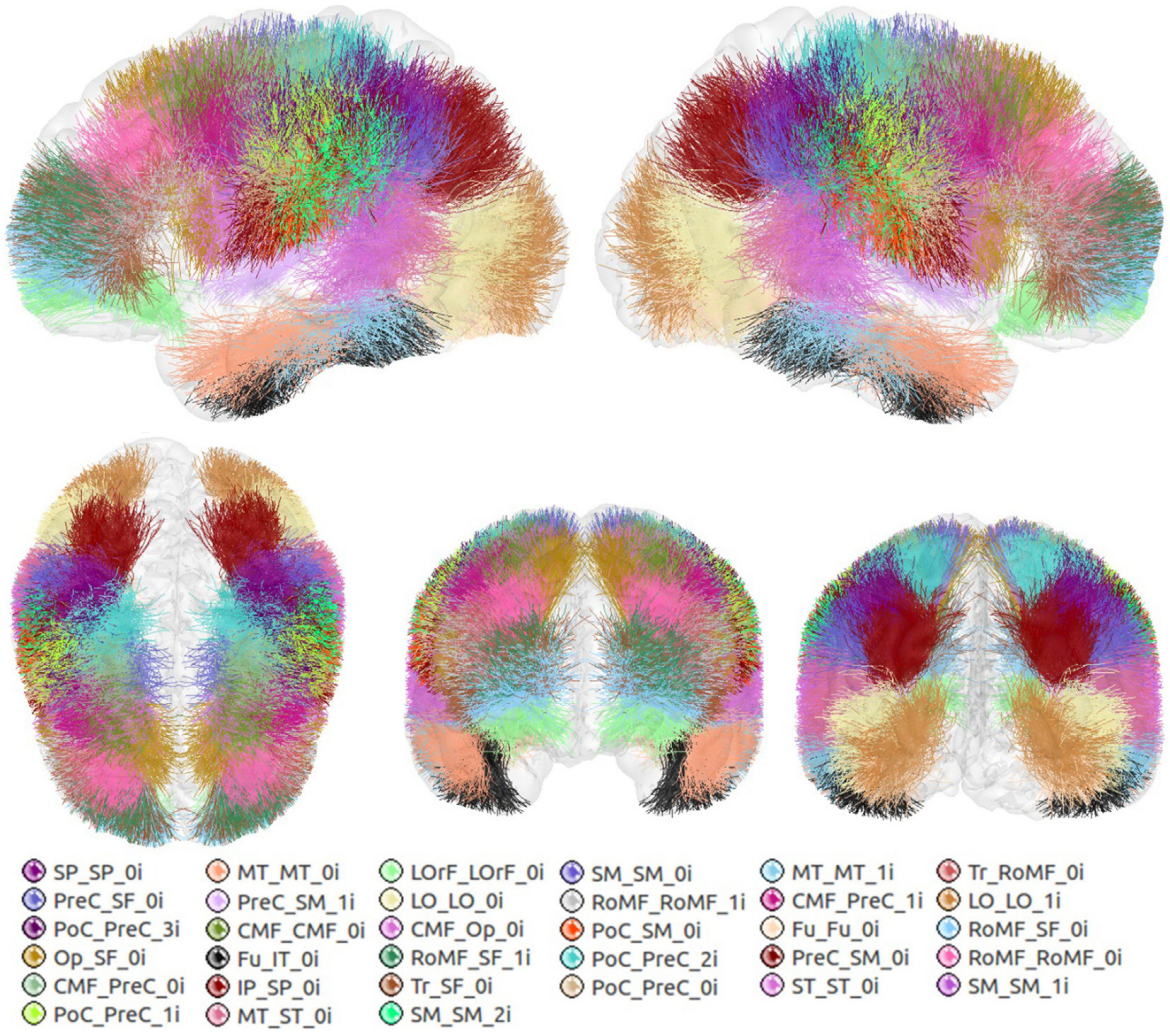
Common bundles between hemispheres and their labels. Lateral, superior, frontal, and posterior views.

**Figure 7:**
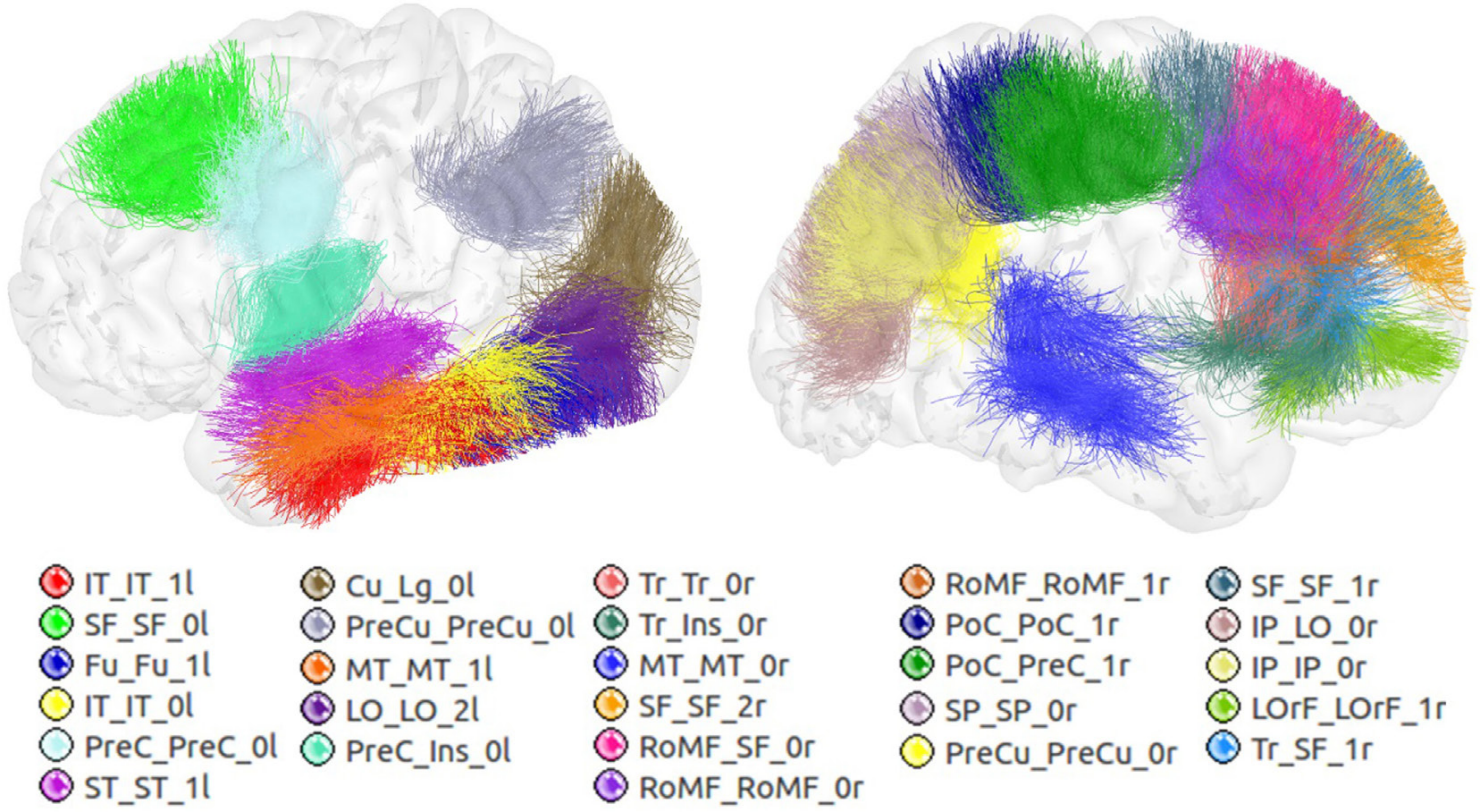
Atlas bundles found only in left or right hemisphere.

**Figure A1:**
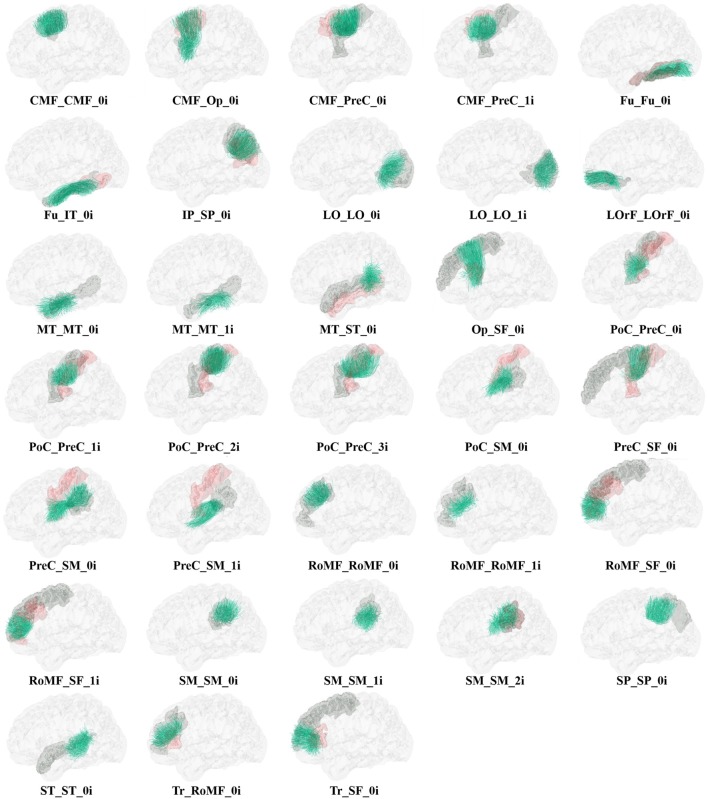
Common bundles between hemispheres and their labels. Separated views.

Figure 6 shows the set of bundles common between hemispheres for the final atlas. Figure A1 in Appendix shows separated views of these bundles. Figure 7 displays the atlas bundles found only in one hemisphere. Tables 5 and 6 list the measure of bundle reproducibility, represented by the number of bootstrap samples including the bundle. Note that these results follow the naming scheme described in Section 2.3.3. A bundle name is defined by the pair of ROIs connected by it. The ROIs used for the labeling are listed in Table 1. A bundle connecting *ROI*_1_ and *ROI*_2_ will be called *ROI*_2__*ROI*_2__*xn*. The number *n* is added to distinguish different bundles connecting the same gyri. The number is assigned randomly from 0 to the maximum number of connections for the pair of ROIs. A letter *x* is finally included to differentiate bundles common to both hemispheres (i), bundles only in left hemisphere (l), and only in right hemisphere (r). For example, a bundle connecting superior frontal and inferior frontal gyri, present in both hemispheres will be called SF_IF_0i. If there exist another bundle connecting these gyri, only in the right hemisphere, it will be called SF_IF_1r.

**Table 5:** Reproducibility of bundles common to both hemispheres, based on the number of bootstrap samples including each bundle.

Label	Left	Right
SP_SP_0i	10	10
PreC_SF_0i	8	10
PoC_PreC_3i	10	10
Op_SF_0i	9	10
CMF_PreC_0i	9	10
PoC_PreC_1i	10	10
MT_MT_0i	9	10
PreC_SM_1i	10	9
CMF_CMF_0i	9	10
Fu_IT_0i	10	10
IP_SP_0i	10	10
MT_ST_0i	10	10
LOrF_LOrF_0i	9	10
LO_LO_0i	10	10
CMF_Op_0i	9	10
RoMF_SF_1i	9	10
Tr_SF_0i	9	10
SM_SM_2i	10	10
SM_SM_0i	9	8
RoMF_RoMF_1i	8	10
PoC_SM_0i	10	10
PoC_PreC_2i	10	10
PoC_PreC_0i	10	10
MT_MT_1i	10	10
CMF_PreC_1i	9	10
Fu_Fu_0i	10	10
PreC_SM_0i	10	10
ST_ST_0i	10	10
Tr_RoMF_0i	9	10
LO_LO_1i	10	10
RoMF_SF_0i	9	10
RoMF_RoMF_0i	8	9
SM_SM_1i	10	10

**Table 6:** Reproducibility for bundles present only in left or right hemisphere, based on the number of bootstrap samples including each bundle.

Left hemisphere		Right hemisphere	
Labels	Left	Labels	Right
IT_IT_1l	10	Tr_Tr_0r	10
SF_SF_0l	9	Tr_Ins_0r	10
Fu_Fu_1l	10	MT_MT_0r	9
IT_IT_0l	8	SF_SF_2r	9
PreC_PreC_0l	9	RoMF_SF_0r	10
ST_ST_1l	9	RoMF_RoMF_0r	10
Cu_Lg_0l	8	RoMF_RoMF_1r	8
PreCu_PreCu_0l	10	PoC_PoC_1r	10
MT_MT_1l	9	PoC_PreC_1r	10
LO_LO_2l	10	SP_SP_0r	10
PreC_Ins_0l	10	PreCu_PreCu_0r	10
		SF_SF_1r	10
		IP_LO_0r	10
		IP_IP_0r	10
		LOrF_LOrF_1r	10
		Tr_SF_1r	9

### 3.3 Automatic Segmentation of SWM Bundles

To test the reproducibility of the SWM bundles of the final atlas, we performed the automatic segmentation of 78 subjects of the segmentation data sets. We calculated the number of subjects where each bundle is present and the variability in the number of fibers. Small bundles with less than 10 fibers were not considered in the analysis.

A lateralization index of the bundle volume was calculated for the 33 common bundles between hemispheres, using the 78 segmented subjects, as described by Catani et al. (2012). The two-tail, unpaired samples, *t*-test was used to evaluate the statistical significance of the volume differences. There were statistically significant differences in only 12 bundles, of which all presented right lateralization (CMF_PreC_1i, LO_LO_1i, MT_MT_0i, MT_ST_0i, PoC_PreC_1i, PoC_PreC_2i, RoMF_RoMF_1i, RoMF_SF_li, SM_SM_0i, SM_SM_2i, SP_SP_0i, and Tr_RoMF_0i).

Table 7 shows the mean lateralization index and the number of subjects correctly segmented for each interhemispheric common bundle. Table 8 shows the number of subjects correctly segmented for the bundles present in only one hemisphere.

**Table 7:** Segmentation results for common bundles between hemispheres: number of segmented subjects and mean lateralization index.

Labels	Num. subjects L	Num. subjects R	Laterality index	Significant differences
*CMF_CMF_*0*i*	77	78	0.040	
*CMF_Op_*0*i*	78	78	0.139	
*CMF_PreC_*0*i*	78	78	0.046	
*CMF_PreC_*1*i*	78	78	0.252	✓
*Fu_Fu_*0*i*	75	77	0.143	
*Fu_IT_*0*i*	76	78	0.190	
*IP_SP_0i*	78	78	−0.004	
*LO_LO_*0*i*	78	78	−0.002	
*LO_LO_*1*i*	75	78	0.349	✓
*LOrF_LOrF_*0*i*	78	75	−0.171	
*MT_MT_*0*i*	78	76	0.282	✓
*MT_MT_*1*i*	70	75	0.207	
*MT_ST_*0*i*	76	76	0.365	✓
*Op_SF_*0*i*	78	77	0.063	
*PoC_PreC_*0*i*	70	73	0.021	
*PoC_PreC_*1*i*	78	78	−0.065	
*PoC_PreC_*2*i*	78	78	0.131	✓
*PoC_PreC_*3*i*	78	78	0.308	✓
*PoC_SM_*0*i*	78	76	0.114	
*PreC_SF_*0*i*	75	75	0.152	
*PreC_SM_*0*i*	78	77	0.154	
*PreC_SM_*1*i*	77	77	0.131	
*RoMF_RoMF_*0*i*	78	76	0.204	
*RoMF_RoMF_*1*i*	76	77	0.389	✓
*RoMF_SF_*0*i*	77	77	0.0350	
*RoMF_SF_*1*i*	76	78	0.35	✓
*SM_SM_*0*i*	77	74	0.276	✓
*SM_SM_*1*i*	75	76	−0.098	
*SM_SM_*2*i*	78	78	0.281	✓
*SP_SP_*0*i*	78	78	0.197	✓
*ST_ST_*0*i*	74	77	0.151	
*Tr_RoMF_*0*i*	75	77	0.354	✓
*Tr_SF_*0*i*	77	77	−0.012	

**Table 8:** Segmentation results for SWM bundles present in only one hemisphere: number of segmented subjects.

Labels	Num. subjects L	Num. subjects R
*Cu_Lg_*0*l*	73	–
*Fu_Fu_*1*l*	69	–
*IT_IT_*0*l*	75	–
*IT_IT_*1*l*	77	–
*LO_LO_*2*l*	78	–
*MT_MT_*1*l*	73	–
*PreC_Ins_*0*l*	77	–
*PreC_PreC_*0*l*	78	–
*PreCu_PreCu_*0*l*	77	–
*SF_SF_*0*l*	73	–
*ST_ST_*1*l*	76	–
*IP_IP_*0*r*	–	78
*IP_LO_*0*r*	–	78
*LOrF_LOrF_*1*r*	–	75
*MT_MT_*0*r*	–	78
*PoC_PoC_*1*r*	–	78
*PoC_PreC_*1*r*	–	78
*PreCu_PreCu_*0*r*	–	77
*RoMF_RoMF_*0*r*	–	78
*RoMF_RoMF_*1*r*	–	78
*RoMF_SF_*0*r*	–	77
*SF_SF_*1*r*	–	76
*SF_SF_*2*r*	–	71
*SP_SP_*0*r*	–	78
*Tr_Ins_*0*r*	–	78
*Tr_SF_*1*r*	–	78
*Tr_Tr_*0*r*	–	77

**Figure 8:**
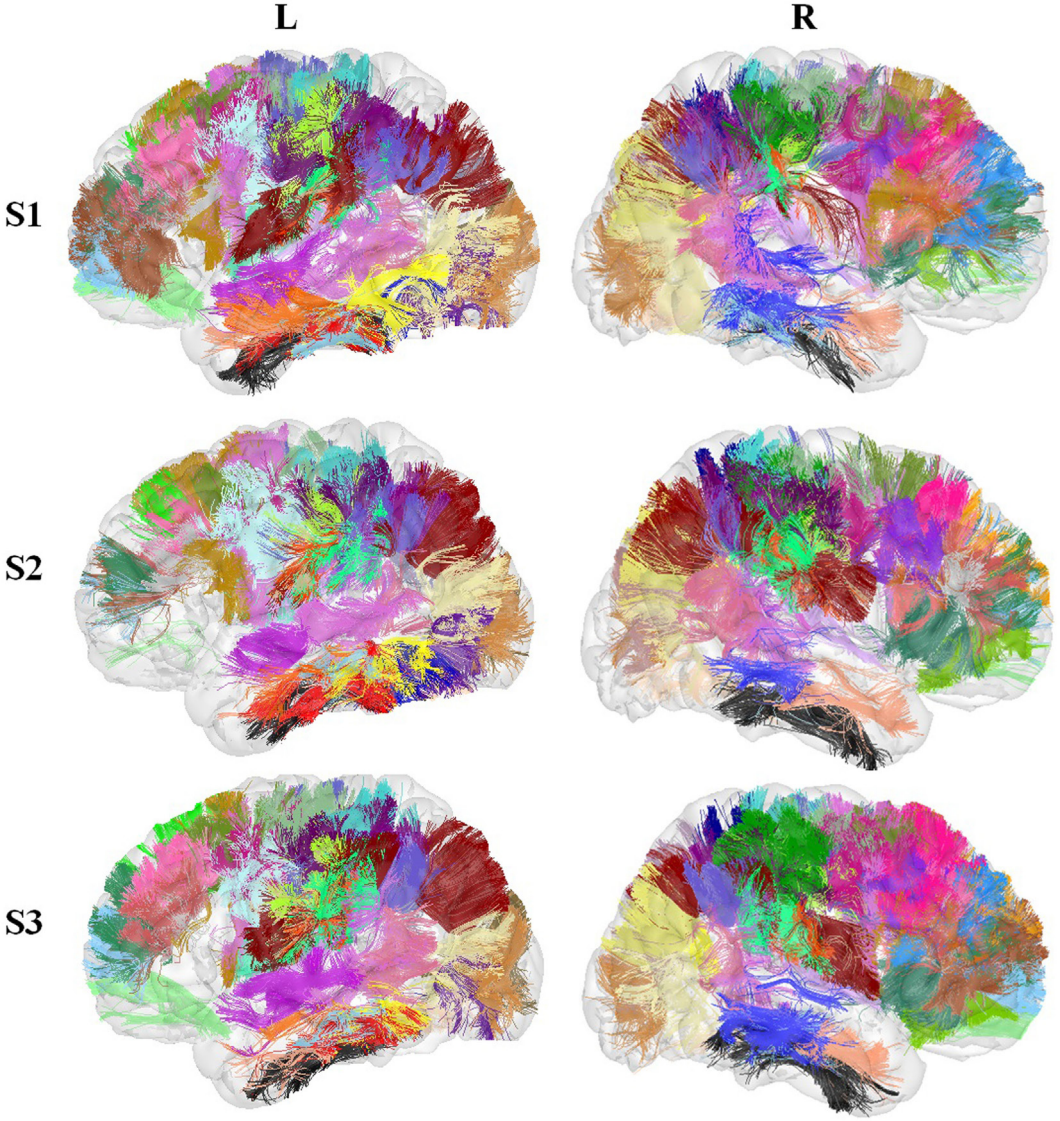
Three subjects segmented using the SWM bundle atlas.

**Figure 9:**
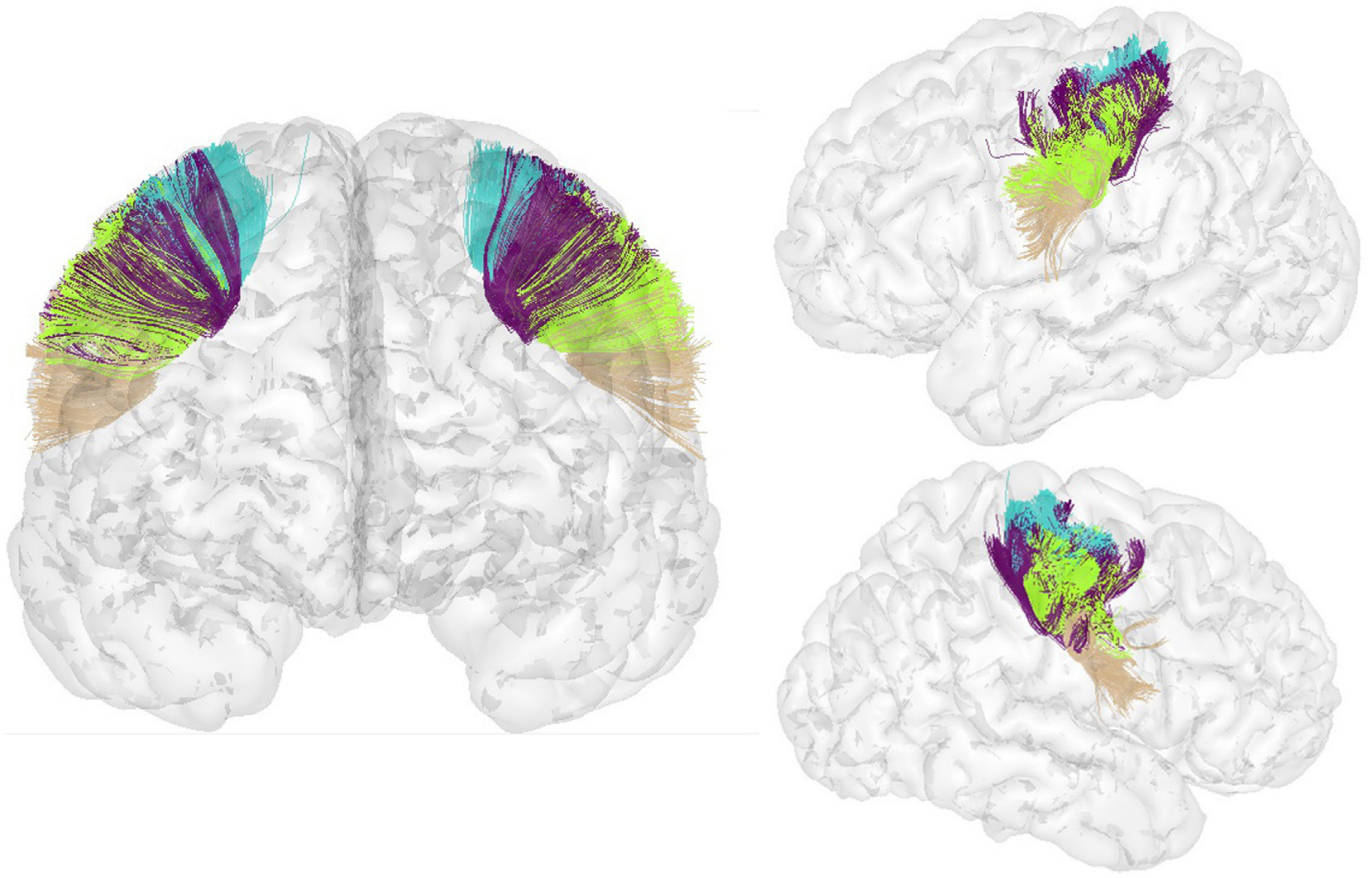
Bundles connecting the precentral and postcentral ROIs in both hemispheres segmented in a new subject.

Figure 8 shows the segmented bundles for 3 of the 78 subjects, for both hemispheres. Figure 9 shows an example of the segmentation results for bundles connecting the precentral and postcentral gyri, for both hemispheres of a single subject.

From Table 7, we can observe that a good reproducibility was obtained for all the atlas bundles, where 46% of the bundles were found in all the subjects. Remaining bundles were missed in some cases, but still present a very high reproducibility. Regarding laterality, some bundles were found to be right lateralized, but further studies must be performed to validate these results.

A comparison between the created SWM bundle atlas and the atlas proposed by Guevara et al. (2017) was performed. The LNAO-SWM79 atlas is composed of 50 bundles per hemisphere, with 35 bundles common to both hemispheres. This atlas was constructed using anatomical information for the extraction of the fibers and fiber shape analysis based on fiber clustering for identification of the bundles. For the comparison, pairs of bundles from both atlases were considered similar if their mean distance (*d_ME_*) was smaller than 15 mm. After the intersection percentage was calculated, 14 bundles with more than 50% of intersection were found for the left hemisphere, and 21 bundles were found for the right hemisphere. Results confirm that most stable bundles were found by both approaches, in particular those from frontoparietal and insula regions. Visual inspection suggests that bundles from our results are more homogeneous, probably due to our use of non-linear registration. This registration method also leads to more reproducible results across subjects, which is the reason why the other study found more, but less reproducible, bundles in some regions. Furthermore, our method could identify a non-negligible number of bundles connecting areas within a single region (21 bundles in the LH and 25 in the RH), which is a feature that was not analyzed by the other approach. The comparison results are presented in Table A1 in Appendix B, where the similar bundles between our results and the LNAO-SWM79 atlas are shown.

## 4 Discussion and Conclusion

We developed and applied an unsupervised method to identify short bundles of the SWM based on the intersubject hierarchical clustering of the whole brain. The algorithm is applied to two groups of N independent subjects in both hemispheres. The goal is to identify superficial bundles present in most of the subjects, representing the connectivity of the human brain short fibers, without the use of anatomical information to guide the segmentation. Our method is fully automatic, avoiding a manual labeling step. Before clustering, the algorithm removes fibers that were portions of known DWM bundles in addition to filtering the fibers according to their length, thus allowing us to perform the clustering with a significantly smaller number of fibers and to automatically discard artifacts. Then, intersubject clustering is performed, identifying the representative bundles present in at least 75% of the population. This reproducibility criterion is very strict compared to those usually described in the literature. Subsequently, an anatomical labeling, based on cortical parcellation, allows us to analyze the most probable connection, thus discarding more suspicious or non-reproducible bundles. Also, interhemispheric correspondence of the bundles is found and used to label common bundles between both hemispheres.

Finally, through intergroup comparison, bundles present in the two different groups are selected, keeping only reproducible bundles to create a robust atlas. The algorithm was first applied using both linear and non-linear registrations to two groups of 37 subjects. Non-linear registration produced better results when using a restrictive threshold of 7 mm as the maximum mean distance between bundles. With non-linear registration, we identified a greater number of similar bundles. In addition, the clusters are more dense and homogeneous, in comparison with those found through linear registration. Therefore, non-linear-registration was selected for the method validation and creation of the final atlas.

A bagging strategy (bootstrap aggregating) was performed over 74 subjects. The robust atlas creation method was repeated 10 times, employing sets of 54 subjects sampled randomly from the database, split into two groups of 27 subjects. A final atlas was constructed by fusing the 10 SWM bundle atlases obtained for each hemisphere.

The final atlas is composed of 44 bundles in the left hemisphere and 49 in the right hemisphere, with a reproducibility ranging from 8/10 to 10/10. These values guarantee that the obtained bundles are representative of the population of subjects and also give a measure of reproducibility of the bundles. For instance, a subset of the most reproducible bundles could be used as first approach, for SWM studies on clinical data.

In general, previous works on short WM fibers have studied bundles connecting two different anatomical regions. Most of the bundles in the frontoparietal regions have been previously described, using both dMRI and postmortem dissection (Catani et al., 2012; Vergani et al., 2014). Since the bundle description presented by Catani et al. (2012) is very precise, we could perform a comparison between the resulting bundles. We can highlight very similar results corresponding to the bundles connecting the following gyri: regions of the superior frontal gyrus with opercularis region of the inferior frontal gyrus (which constitute the frontal aslant tract), regions of the superior frontal gyrus with the middle frontal gyrus, a region of the middle frontal and precentral gyri, several bundles connecting the precentral and postcentral gyrus, and bundles connecting the insula with neighboring regions (triangularis and precentral gyrus). In addition, this work also described some short fibers of the frontal superior longitudinal and frontal inferior longitudinal tracts, also present in the proposed atlas. From the study by Vergani et al. (2014) results, we found the bundle connecting the supplementary motor area, located in the posterior part of the superior frontal gyrus, with the precentral gyrus. Another interesting tractography study is the one proposed by Zhang et al. (2010). Even though no detailed description of the short bundles was provided, the existence of fibers connecting two regions was reported for the whole brain. In addition to the bundles mentioned above, this analysis listed the presence of the bundles connecting the following gyri, also included in our atlas: middle frontal and inferior frontal gyrus, middle frontal and precentral gyrus, cuneus and lingual gyrus, supramarginal gyri and postcentral gyri, and superior temporal and middle temporal gyri. However, no postmortem validation has been performed for those bundles. Furthermore, our method could find a non-negligible number of bundles connecting the same gyri. One example are the connections within the precentral gyrus, which have been previously described in Magro et al. (2014), using tractography and postmortem dissection.

This is the first work that proposes a method based on whole-brain white matter fiber clustering for the study of short WM bundles. Even though several algorithms have been proposed for the clustering of white matter tracts, those have not been tested with short fibers. Our experience tells that algorithms that consider a mean distance between fibers and those that transform the fibers to a low-dimensional space, like spectral clustering, could be less discriminative with small short fiber bundle differences. The main advantage of the proposed method is that it does not rely on automatic anatomical labeling for the main fiber analysis and uses a whole-brain clustering strategy. The method only identifies the most reproducible connections, indistinctly, between two regions or within a single region. In addition, this is, to the best of our knowledge, the first work that compares linear and non-linear registration results. This factor is especially important for superficial white matter, which is known to be more variable across subjects. Thanks to the use of non-linear registration, the proposed atlas contains more reproducible, homogeneous, and dense bundles. Compared to a recent SWM reproducibility study (Guevara et al., 2017), our method could identify most of the stable connections and, as mentioned above, a large number of new bundles that mainly connect two areas of the same region. From computational efficiency point of view, our algorithms run on a standard workstation with reasonable execution times. Moreover, further optimizations such as the work described in the study by Labra et al. (2017) can be used to boost performance by exploiting the parallelism available in current and ubiquitous multicore processors and graphical processing units.

The proposed clustering method is limited by the total number of centroids that can be analyzed at the same time. We tested our algorithm with a maximum of 150,000 centroids. However, as we use preclustered data, the total number of fibers that can be analyzed is notably higher, considering that this preprocessing typically converts a data set of one million fibers into about 5,500 centroids. Another potential limitation is the use of similar parameters for all the brain regions, but since we do not use the number of clusters as a parameter, the algorithm naturally finds more clusters in regions where more structured bundles can be identified. Also, it would be possible to normalize the similarity measure relative to the region where the fibers are from, but the criterion to be used could be difficult to define. Other limitations are common to all neuroimaging analyzes, for example, the dependency to the registration strategy. As we have shown in this work, non-linear registration leads to better results, but further work is required to compare alternative registration strategies.

Other limitations stem directly from diffusion modeling and tractography techniques. Current tractography approaches strive to solve an ill-posed computational problem that can produce reproducible false-positive bundles (Maier-Hein et al., 2016). Hence, some bundles found by our method could be artifacts. As described above, some short association bundles have already been validated by postmortem dissections, but in the future, other techniques could help to increase our knowledge of WM structure and organization. This information could also be used to improve tractography algorithms. The non-existence of a ground truth also limits the method optimizations and validations that can be performed.

A preliminary analysis was also performed, using the created atlas, for the automatic segmentation of 78 new subjects. We could confirm the reproducibility of the selected short bundles. Also, a lateralization analysis was executed, using the segmented bundle volume. No lateralization was found for most of the bundles, with a few of them presenting a slight right lateralization. Further analyzes must be carried out in the future to extend and improve these results. A potential source of uncertainty is the segmentation method, which could be specially adapted for short association bundles by taking into account more information, like a more detailed description of fiber shape or a sulci segmentation.

In conclusion, the proposed method allows the identification of stable and reproducible short bundles across subjects for the whole brain, using an intersubject clustering.

## Author Contributions

CR (main author): algorithm development, results evaluation, and article writing. MG: algorithm development, results evaluation, and article writing. RV: optimization of algorithms and data testing. MF: optimization of algorithms and review of the entire manuscript. JH: database generation, database preprocessing, and results evaluation. DD: development of tools for tractography data processing and database generation. CP: development of tools for tractography data processing, database generation, and results evaluation. J-FM: development of tools for tractography data processing, database generation, results evaluation, and review of the entire manuscript. PG: algorithm development, results evaluation, article writing, and review of the entire manuscript.

## Conflict of Interest Statement

The authors declare that the research was conducted in the absence of any commercial or financial relationships that could be construed as a potential conflict of interest.

## Footnotes

^1^http://nipy.sourceforge.net/nipy.

^2^http://surfer.nmr.mgh.harvard.edu/.

^3^http://dti-tk.sourceforge.net/.

## Appendix B

### Comparison with LNAO-SWM79 Atlas

**Table A1:** Comparison between our results and the LNAO-SWM79 atlas.

LH		RH	
Label	Intersection	Label	Intersection
*CMF_Op_*0*i*	>50%	*CMF_CMF_*0*i*	>50%
*CMF_PreC_*0*i*	<20%	*CMF_Op_*0*i*	<20%
*CMF_PreC_*1*i*	<20%	*CMF_PreC_*0*i*	<20%
*Fu_Fu_*1*l*	<20%	*CMF_PreC_*1*i*	>50%
*IP_SP_*0*i*	>50%	*Fu_Fu_*0*i*	<20%
*IT_IT_*0*l*	<20%	*IP_LO_*0*r*	>50%
*LO_LO_*0*i*	20–50%	*IP_SP_*0*i*	>50%
*LO_LO_*2*l*	<20%	*LO_LO_*0*i*	20–50%
*LOrF_LOrF_*0*i*	20–50%	*LO_LO_*1*i*	<20%
*MT_ST_*0*i*	<20%	*LOrF_LOrF_*0*i*	<20%
*Op_SF_*0*i*	>50%	*LOrF_LOrF_*1*r*	>50%
*PoC_PreC_*0*i*	>50%	*MT_MT_*0*r*	<20%
*PoC_PreC_*1*i*	>50%	*MT_MT_*1*i*	>50%
*PoC_PreC_*2*i*	>50%	*MT_ST_*0*i*	20–50%
*PoC_PreC_*3*i*	<20%	*Op_SF_*0*i*	>50%
*PoC_SM_*0*i*	>50%	*PoC_PoC_*1*r*	>50%
*PreC_Ins_*0*l*	>50%	*PoC_PreC_*0*i*	<20%
*PreC_PreC_*0*l*	>50%	*PoC_PreC_*1*i*	>50%
*PreC_SF_*0*i*	<20%	*PoC_PreC_*1*r*	<20%
*PreC_SM_*0*i*	>50%	*PoC_PreC_*2*i*	>50%
*PreC_SM_*1*i*	>50%	*PoC_PreC_*3*i*	>50%
*PreCu_PreCu_*0*l*	<20%	*PoC_SM_*0*i*	<20%
*RoMF_SF_*0*i*	>50%	*PreC_SM_*0*i*	>50%
*RoMF_SF_*1*i*	<20%	*PreC_SM_*1*i*	>50%
*SM_SM_*0*i*	<20%	*PreCu_PreCu_*0*r*	<20%
*SM_SM_*2*i*	<20%	*RoMF_RoMF_*0*i*	<20%
*SP_SP_*0*i*	>50%	*RoMF_RoMF_*0*r*	<20%
*ST_ST_*0*i*	<20%	*RoMF_RoMF_*1*i*	<20%
*ST_ST_*1*l*	<20%	*RoMF_RoMF_*1*r*	<20%
*Tr_SF_*0*i*	>50%	*RoMF_SF_*0*i*	>50%
		*RoMF_SF_*0*r*	>50%
		*RoMF_SF_*1*i*	<20%
		*SM_SM_*0*i*	<20%
		*SM_SM_*2*i*	>50%
		*SP_SP_*0*i*	>50%
		*SP_SP_*0*r*	<20%
		*ST_ST_*0*i*	>50%
		*Tr_Ins_*0*r*	>50%
		*Tr_SF_*0*i*	>50%
		*Tr_SF_*1*r*	<20%
		*Tr_Tr_*0*r*	>50%
